# The effects of non-pharmaceutical interventions on SARS-CoV-2 transmission in different socioeconomic populations in Kuwait: a modeling study

**DOI:** 10.1186/s12889-021-10984-6

**Published:** 2021-05-26

**Authors:** Fatima Khadadah, Abdullah A. Al-Shammari, Ahmad Alhashemi, Dari Alhuwail, Bader Al-Saif, Saud N. Alzaid, Barrak Alahmad, Isaac I. Bogoch

**Affiliations:** 1 Department of Medicine, University of Toronto, Toronto, ON Canada; 2 Division of Medical Oncology and Hematology, Princess Margaret Cancer Centre, 610 University Ave, 700U 6W458, Toronto, ON M5G 2M9 Canada; 3 Department of Mathematics, Faculty of Sciences, Kuwait University, Khaldiya, Kuwait; 4 Dasman Diabetes Institute, Dasman, Kuwait; 5 Department of Medicine, Adan Hospital, Ministry of Health, Al-Ahmadi, Kuwait; 6 Department of Information Science, College of Life Sciences, Kuwait University, Sabah Al-Salem University City, Sabah Al-Salem, Kuwait; 7 Department of History, College of Arts, Kuwait University, Sabah Al-Salem University City, Sabah Al-Salem, Kuwait; 8 Carnegie Middle East Center, Beirut, Lebanon; 9 Department of Surgery, Faculty of Medicine, Kuwait University, Jabriya, Kuwait; 10 Department of Environmental Health, Harvard T.H. Chan School of Public Health, Harvard University, Boston, MA USA

**Keywords:** COVID-19, Non-pharmaceutical interventions, Mathematical modeling, Socioeconomic disparities

## Abstract

**Background:**

Aggressive non-pharmaceutical interventions (NPIs) may reduce transmission of SARS-CoV-2. The extent to which these interventions are successful in stopping the spread have not been characterized in countries with distinct socioeconomic groups. We compared the effects of a partial lockdown on disease transmission among Kuwaitis (P_1_) and non-Kuwaitis (P_2_) living in Kuwait.

**Methods:**

We fit a modified metapopulation SEIR transmission model to reported cases stratified by two groups to estimate the impact of a partial lockdown on the effective reproduction number (${\mathcal{R}}_e$). We estimated the basic reproduction number (${\mathcal{R}}_0$) for the transmission in each group and simulated the potential trajectories of an outbreak from the first recorded case of community transmission until 12 days after the partial lockdown. We estimated ${\mathcal{R}}_e$ values of both groups before and after the partial curfew, simulated the effect of these values on the epidemic curves and explored a range of cross-transmission scenarios.

**Results:**

We estimate ${\mathcal{R}}_e$ at 1·08 (95% CI: 1·00–1·26) for P_1_ and 2·36 (2·03–2·71) for P_2_. On March 22nd, ${\mathcal{R}}_e$ for P_1_ and P_2_ are estimated at 1·19 (1·04–1·34) and 1·75 (1·26–2·11) respectively. After the partial curfew had taken effect, ${\mathcal{R}}_e$ for P_1_ dropped modestly to 1·05 (0·82–1·26) but almost doubled for P_2_ to 2·89 (2·30–3·70). Our simulated epidemic trajectories show that the partial curfew measure greatly reduced and delayed the height of the peak in P_1_, yet significantly elevated and hastened the peak in P_2_. Modest cross-transmission between P_1_ and P_2_ greatly elevated the height of the peak in P_1_ and brought it forward in time closer to the peak of P_2_.

**Conclusion:**

Our results indicate and quantify how the same lockdown intervention can accentuate disease transmission in some subpopulations while potentially controlling it in others. Any such control may further become compromised in the presence of cross-transmission between subpopulations. Future interventions and policies need to be sensitive to socioeconomic and health disparities.

**Supplementary Information:**

The online version contains supplementary material available at 10.1186/s12889-021-10984-6.

## Background

On February 20th, with zero reported cases of coronavirus disease 2019 (COVID-19) in Kuwait and only two reported deaths in neighboring Iran [1], the State of Kuwait ordered immediate closure of its shared borders and subsequent evacuation of its citizens from Iran [2]. The first group of evacuees from Mashhad, Iran were asked to self-isolate at home while the second group of evacuees from Tehran and Qom were placed in institutional quarantine. Estimates put the true number of cases in Iran at the time to be in the range of 18,300 (95% confidence interval: 3770 to 53,470) [3]. This intervention was subsequently followed by further border control measures, closures of schools, non-essential businesses and public gathering spots, cessation of all commercial flights and, eventually a partial lockdown on March 22nd 2020 [4–6].

Since the beginning of the epidemic, without effective pharmaceutical interventions available to prevent or treat COVID-19, countries largely rely on non-pharmaceutical interventions (NPIs) to reduce disease transmission [7, 8]. Early combined NPIs were shown to reduce disease transmission and delay peak death rates. Additionally, timing of NPI implementation appears to correlate with peak death rates [8]. Nations which were able to implement early containment measures such as Taiwan, Singapore, Japan and Hong Kong all seemingly were flattening the epidemic curve in the first few months of the pandemic [9]. Despite lags in testing, it was thought that Kuwait would pursue a similar trajectory due to its aggressive early action.

Evidence is accumulating that viral spread is unequal and preys preferentially on lower socioeconomic classes [10–12], but it is unclear how NPIs implemented in various countries are playing a role in this regard. Models of disease transmission thus far have been homogenous and have not yet accounted for these important heterogeneities [13]. Kuwait and the rest of the Gulf States have a unique demographic profile. Nearly 70% of the 4·8 million people living in Kuwait are non-Kuwaiti, and largely represent migrant workers of lower socioeconomic status. The profile of the Non-Kuwaiti population is predominantly male (69%), poorly educated (68% below secondary level education), and relatively young (median age group 30–34) [14] (Table 1).

**Table 1:** Socioeconomic differences between Kuwaiti and non-Kuwaiti residents in Kuwait in 2019

	Kuwaitis (P_**1**_)	Non-Kuwaitis (P_**2**_)
**Population** - N (%)	1,432,045 (30.0)	3,344,362 (70.0)
**Average Monthly Income in Public Sector** [15, 16] - Kuwaiti Dinar (USD)		
Female	1279 (4135)	666 (2153)
Male	1807 (5841)	726 (2347)
**Average Monthly Income in Private Sector** [15, 16] - Kuwaiti Dinar (USD)		
Female	866 (2799)	387 (1251)
Male	1417 (4581)	271 (876)
**Education** - N (%)		
Below secondary school	521,699 (36.4)	2,265,394 (67.7)
Secondary school and above	481,407 (33.6)	552,725 (16.5)
Unknown	428,939 (30.0)	52,6243 (15.7)
Occupation^*^ **-** N (%)		
Manual work	21,288 (4.0)	1,148,897 (44.4)
Non-manual work	568,843 (96.2)	1,291,496 (50.0)
Not stated	1354 (0·2%)	145,865 (5·6%)
**Healthcare Access**	Free medical, dentistry and pharmacy care	Fee-for-service
**Social Welfare**	Available	Not available
**Public Housing**	Subsidized	Not available

^*^Occupations as per International Standard Classification of Occupations. Grouped under manual work are agriculture and fishery workers, craft and related trades work, production monitoring and elementary occupations. Under non-manual are managers, professionals, associate professionals, clerk and sales services workers. Population, education and income data sourced from PACI [14]

In this study, we quantify the differences in viral transmission dynamics of SARS-CoV-2 between Kuwaiti nationals and non-Kuwaitis living in Kuwait using nationality as a surrogate for socioeconomic status. We also look at how NPIs affected transmission dynamics in each group.

## Methods

To explore the impact of heterogeneities in transmission dynamics in Kuwait, we fitted a metapopulation transmission dynamic model to a dataset on reported cases stratified by two socioeconomic groups (Table 1 and Fig. S1a and b in the Supplementary Material). The dataset contained daily numbers of reported confirmed cases from the two groups.

Publicly-available data on COVID-19 cases was collected daily from verified and official government sources [15, 17]. The dataset included cases from the first reported case of COVID-19 on February 24th, 2020 until May 12th, 2020. The model was fitted to case numbers up to April 20th, 2020 in order to disentangle the impact of the partial curfew from other drastic interventions that were implemented past this date. Information was limited to the number of confirmed cases, travel history, nationality, critical cases, recoveries, and deaths.

Information about population density, residential units (houses and apartments) and occupation were obtained from the online Statistics Service System at the Public Authority for Civil Information (PACI); last updated on 21 December, 2019 [14]. To create maps depicting the geographic density of the two populations as well as the residential units we used ArcGIS Pro version 2.5.1 by Esri Inc. The district polygons were obtained from OpenStreetMaps [18].

### Model

**Fig. 1:**
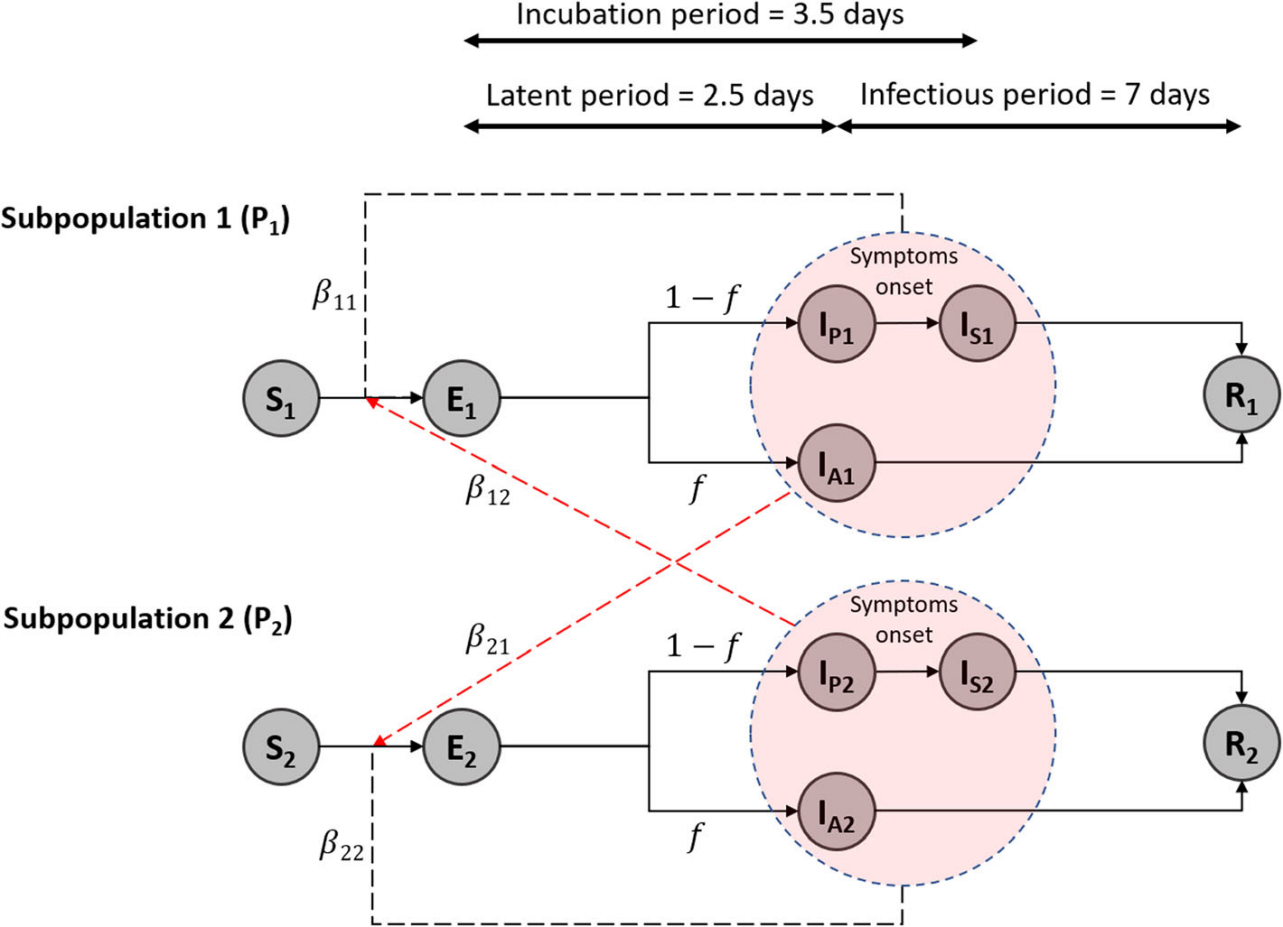
Structure of the modified metapopulation SEIR model. The population is divided into two socioeconomic groups: Subpopulation 1 (P_1_) and Subpopulation 2 (P_2_). Individuals within each subpopulation are divided into the following infection classes or compartments: susceptible (S), exposed but not infectious (E), asymptomatic infectious (I_A_), pre-symptomatic infectious (I_P_), symptomatic infectious (I_S_), and removed (R). The progression through the different compartments is described by key epidemiological timescales that characterize the transmission dynamics of COVID-19 infection

The modified metapopulation SEIR model divides the population into two distinct groups: Subpopulation 1 (P_1_) of presumed higher socioeconomic status and Subpopulation 2 (P_2_) of presumed lower socioeconomic status, who are vastly overrepresented by non-Kuwaitis (Table 1). We analyze the disease transmission by using a modified SEIR model that describes the epidemiological characteristics of COVID-19. The model divides individuals within each subpopulation into the following compartments: susceptible (S), exposed but not infectious (E), asymptomatic infectious (I_A_), pre-symptomatic infectious (I_P_), symptomatic infectious (I_S_), and removed (R). The progression through the different compartments is described by key durations that are known to characterize the infection transmission dynamics of COVID-19 (Table A1 in Supplementary Material). Our model structure is presented in Fig. 1 and details about the model equations and its parametrization are presented in the Supplementary Material.

Given that the population is almost fully susceptible early in the outbreak (due to a relatively small number of cases), we assume the transmission rate for each subpopulation, *β*, is only affected by the implementation of a partial lockdown during this period. Hence, we assume that the transmission rate is a function of time *β*(*t*) = *κ*(*t*)*β*_0_, where *β*_0_ is the baseline transmission rate (i.e. without a partial lockdown) and *κ*(*t*) is a positive step function that models the scaling by which a control measure may reduce (*κ* < 1) or increase (*κ* > 1) the transmission rate:

$$ \kappa (t)=\left\{\begin{array}{c}1,\kern0.5em Before\ lockdown\\ {}\kappa, \kern0.5em During\ lockdown\end{array}\right. $$

We describe the interaction between the two subpopulations using two parameters, *β*_12_ and *β*_21_, to model the successful transmission occurring upon cross-contact between individuals of P_1_ and P_2_. The average interaction between socioeconomic groups is modeled by a 2 × 2 transmission matrix, which describes the disease transmission between and within subpopulations:

$$ \left(\begin{array}{cc}{\beta}_{11}& {\beta}_{12}\\ {}{\beta}_{21}& {\beta}_{22}\end{array}\right) $$

This transmission matrix provides a convenient means for capturing the mixing between and within the subpopulations, with each entry value *β*_*ij*_ modeling the average transmission of the disease from individuals in subpopulation *j* to individuals in subpopulation *i*. In this formulation, we assume that there exists an epidemiological cross-coupling between the two socioeconomic groups with negligible cross-migration. The mixing pattern is assumed to be strongly assortative to reflect the fact that 1) individuals interact strongly and preferentially within their socioeconomic group and 2) migrant workers reside in separate geographical locations. In particular, migrant workers are assumed to only make short-lived visits to work in areas populated by the Kuwaiti subpopulation. These patterns dictate that the between-subpopulation transmission parameters *β*_12_ and *β*_21_ (cross-transmission rates) are considerably smaller than the within-subpopulation transmission parameters *β*_11_ and *β*_22_. Further, individuals from both subpopulations are assumed to have equal cross-contact rates and response to the infection which, in turn, dictates that the between-subpopulation interaction is symmetric, i.e. *β*_12_ = *β*_21_. For convenience, these cross-transmission rates are taken as free adjustable parameters to explore their effect on peak and cumulative incidence of COVID-19 infections, with our model results checked for sensitivity (see Table A1). The other transmission rates, *β*_11_ and *β*_22_, capture the disease dynamics within subpopulations and are assumed to reflect the bulk of the early transmission dynamics in Kuwait where the observed social mixing patterns dictate that early transmission is effectively decoupled. Hence, the impact of the partial lockdown is expected to be entirely captured by changes to the baseline values of these within-subpopulation transmission rates. We model these changes by allowing each subpopulation to have its own scaling factor, *κ*, which we estimate by fitting our model to the numbers of early confirmed cases in each subpopulation.

We assume the outbreak started in both subpopulations with a single infectious case on February 24, 2020 and with both subpopulations being initially susceptible. To capture the initial heterogeneity in contact structure, we estimate a basic reproduction number (${\mathcal{R}}_0$) for each subpopulation by fitting their early outbreak data to an individual modified SEIR model [19]. Similarly, we quantify the impact of imposing a partial lockdown on March 22, 2020 by estimating the effective reproduction number (${\mathcal{R}}_e$) for each subpopulation during the lockdown period. We note here that the impact of the partial lockdown is inherently characterized by the scaling factor *κ*, where ${\mathcal{R}}_e=\kappa {\mathcal{R}}_0$. In other words, *κ* plays the role of an effectiveness parameter for the intervention, with values less than 1 characterizing the intervention as effective.

Our results for both reproductive numbers are directly derived from the estimation of two unknown model parameters for each subpopulation: *β*_0_ and *κ*. In short, we derive maximum likelihood estimates (MLE) of these unknown parameters by assuming the observed numbers of reported cases follow a negative binomial process, thus allowing for estimates of model uncertainty. Optimization was carried out using the Nelder-Mead method and parameter uncertainty was represented by quantile-based 95% confidence intervals (CI). Our estimates of the baseline and effective transmission rates are then used to simulate the epidemic curves of each subpopulation and under various cross-transmission scenarios. All of our simulations, parameter estimation and model fitting were run in the R software environment [20] as described elsewhere [19].

In Kuwait, COVID-19 transmission outside of the home is mainly observed in healthcare facilities and essential businesses. We assume that these are the places where transmission occurs from individuals living in COVID-19 reservoirs (P_2_) to those not living there (P_1_). We explore the effect of this cross-transmission by simulating the epidemic curves for *β*_12_ = *β*_21_ = 0.01 or 0.02 . Here *β*_12_ = 0 models the situation where isolation of infection reservoirs is stringent. Increasing this value simulates the situation where some individuals from P_2_ are allowed to work in healthcare facilities and essential businesses.

The data and codes that support the findings of this study are fully available from the corresponding author, upon reasonable request.

## Results

We present our estimates of the reproduction numbers in Table 2. The basic reproduction numbers for P_1_ (${\mathcal{R}}_{0,1}$) and P_2_ (${\mathcal{R}}_{0,2}$) are 1·08 (95% CI: 1·00–1·26) and 2·36 (2·03–2·71), respectively. Prior to the partial curfew, the effective reproduction numbers were 1·19 (1·04–1·34) and 1·75 (1·26–2·11) for P_1_ and P_2_, respectively. Imposing a partial curfew greatly reduced the reproduction number to 1·05 (0·82–1·26) in P_1_, while significantly increasing it to 2·89 (2·30–3·70) in P_2_. These results suggest that the effects of lockdowns vary across socioeconomic groups. After imposing additional geographic isolation on P_2_ combined with focused testing, contact-tracing and isolation the effective reproduction number was reduced to 1·51 (1·13–1·96). Over this time period, death rates for P_1_ were 5·59 per million inhabitants, compared to 9·57 per million inhabitants for P_2_.

**Table 2:** Basic and effective reproduction numbers for P_1_ and P_2_ (higher and lower socioeconomic groups respectively)

	Effective reproduction number, ${\mathcal{R}}_e$			Basic reproduction number, ${\mathcal{R}}_0$
Subpopulation	25 Feb – 22 Mar (Prior to partial lockdown)	22 Mar – 3 Apr (After partial lockdown)	19-Apr-2020	
P_1_	1·19 (1·04–1·34)	1·05 (0·82–1·26)	1·02 (0·91–1·14)	1·08 (1·00–1·26)
P_2_	1·75 (1·26–2·11)	2·89 (2·30–3·70)	1·51 (1·13–1·96)	2·36 (2·03–2·71)

* 95% Confidence Interval (CI) values are given in parentheses. P_1_ and P_2_ are the subpopulations of higher and lower socioeconomic status, respectively

**Fig. 2:**
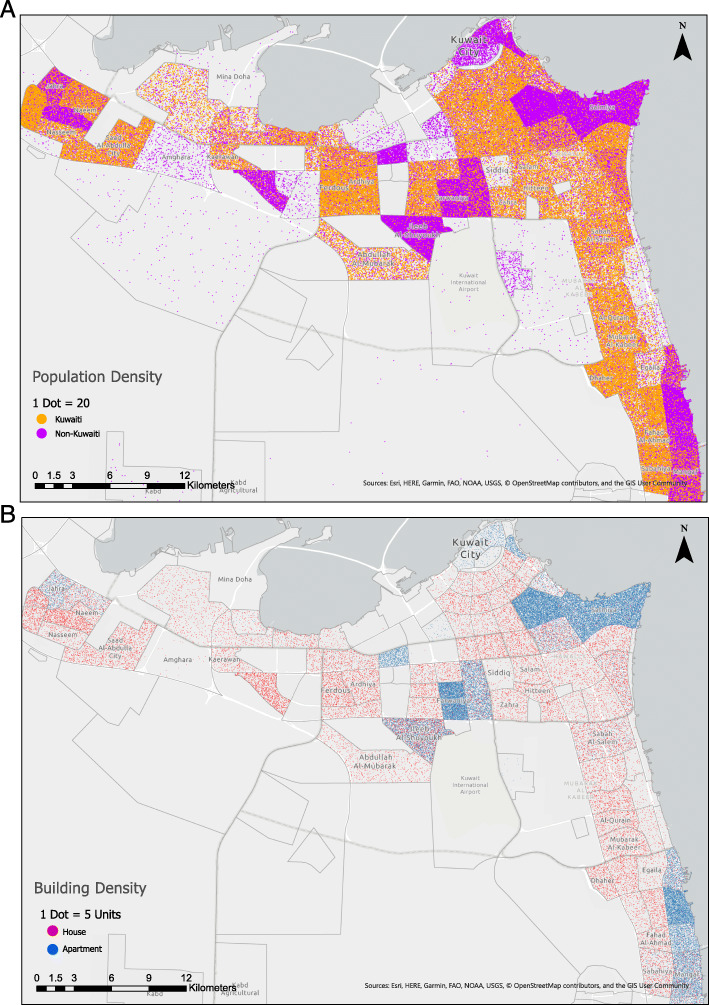
Spatial structure of the population in Kuwait. **a** A geographical density distribution of the Kuwaiti and Non-Kuwaiti subpopulations. **b** A spatial density of the housing buildings by districts in Kuwait

**Fig. 3:**
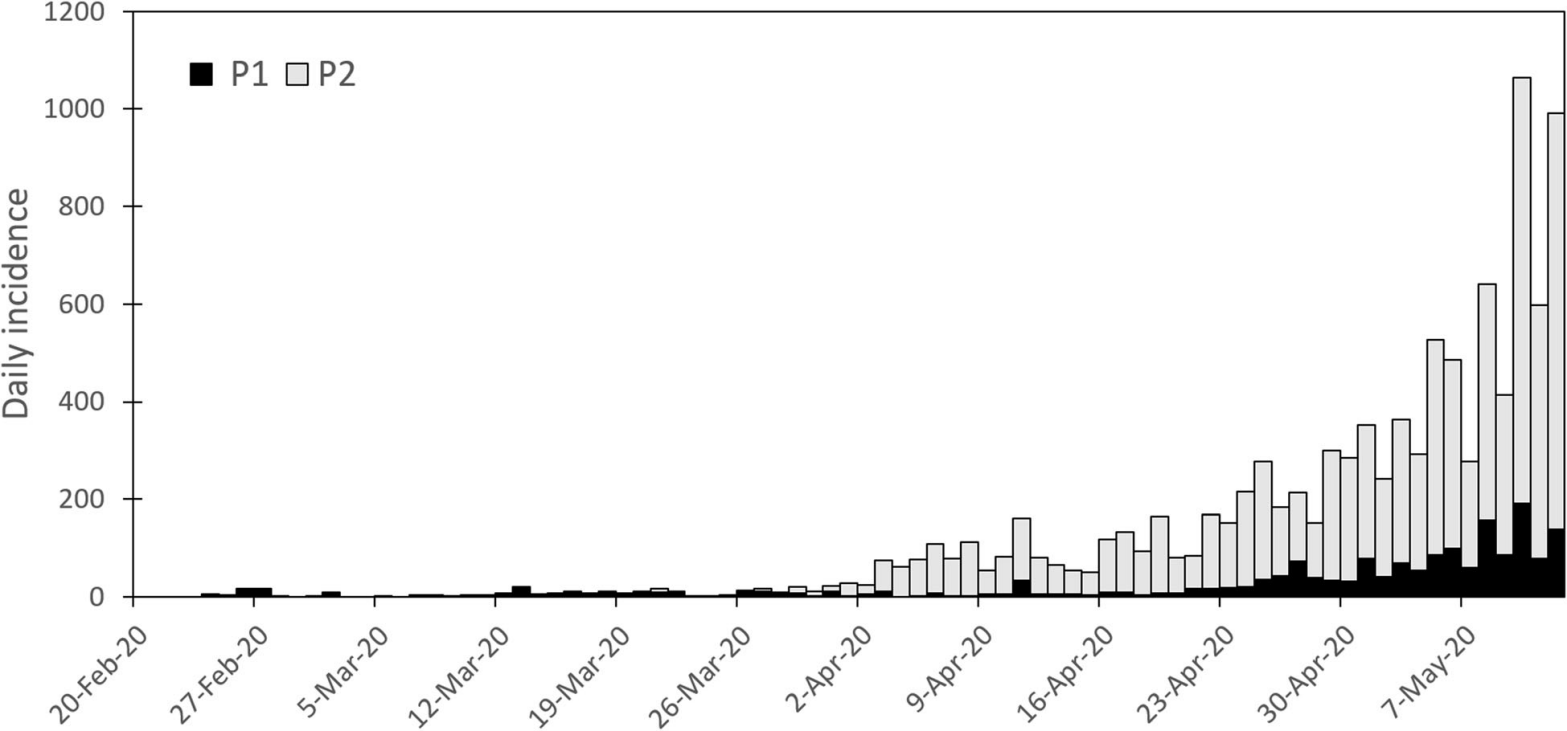
Temporal dynamics of COVID-19 in Kuwait from Feb-May 2020

Figure 2a and b shows the subpopulation and building densities in the country. Areas of increased population density, in particular that of the non-Kuwaiti population correlate geographically with increased housing density and are almost interposable. Figure 3 highlights the daily number of cases before and after the institution of the lockdown in the two populations.

**Fig. 4:**
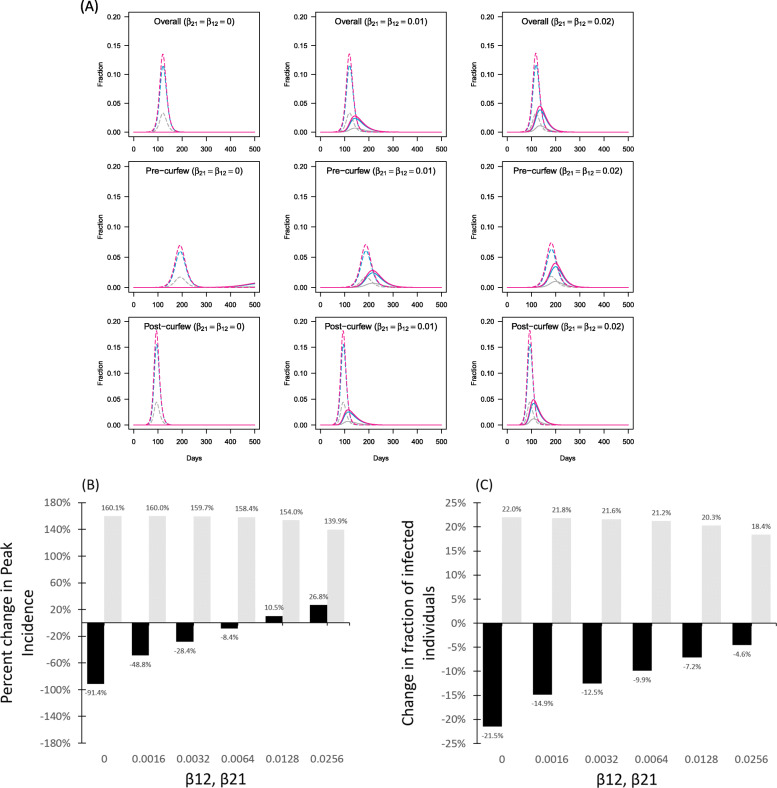
**a** Effect of partial curfew on the epidemic curves. Model simulations of the daily infection incidence for P_1_ and P_2_ (higher and lower socioeconomic groups respectively) before and after the partial curfew. (Overall) Simulations based on the basic reproduction numbers ${\mathcal{R}}_{0,1}=1\cdotp 08$ and ${\mathcal{R}}_{0,2}=2\cdotp 36$. (Pre-curfew) Simulations based on pre-curfew reproduction numbers ${\mathcal{R}}_{e,1}=1\cdotp 19$ and ${\mathcal{R}}_{e,2}=1\cdotp 75$. (Post-curfew) Simulations based on post-curfew reproduction numbers ${\mathcal{R}}_{e,1}=1\cdotp 05$ and ${\mathcal{R}}_{e,2}=2\cdotp 89$. Cross-transmission is modeled by using *β*_12_ = *β*_12_ = 0, 0 · 01, 0 · 02 to simulate an increase in uncontrolled transmission (or leak) from individuals in P_2_ to P_1_ and vice versa. Solid lines refer to P_1_ and dashed lines refer to P_2_. Line colors refer to different infection states: Grey = asymptomatic infections, pink = pre-symptomatic & symptomatic infections, and blue = symptomatic infections. **b-c** Change in the peak and overall infections of COVID-19 in P_1_ and P_2_ under different cross-transmission scenarios. From left to right, with increasing symmetric cross-transmission between P_2_ and P_1_ (i.e. *β*_12_ = *β*_12_), less and less cases are averted in P_1_ and the effect of the NPI on peak incidence is gradually blunted. The maximum averted COVID-19 infections in P_1_ is found in the absence of cross-transmission. In this case, the peak incidence is reduced by 91.4% and the total infections are averted by 21.5%. Black color refers to P_1_ and grey color refers to P_2_. (Vertical axes have been amended to highlight the differences in the effect of the intervention)

Simulations based on these reproduction numbers model the effect of a partial curfew on the epidemic curves (Fig. 4). Our simulations show that the two subpopulations had distinct daily incidence dynamics prior to the partial curfew, with P_1_ showing a significantly lower and delayed peak incidence (about 8 times lower than P_2_). After imposing the partial curfew, the two peaks diverge further with P_2_ experiencing a significantly higher and earlier peak than P_1_ (more than 200 times higher than P_1_). In particular, the partial curfew is predicted to have had the effect of averting 21.5% of the total COVID-19 infections and reducing the peak incidence by 91.4% in subpopulation 1 (Figs. 4b and c). In contrast, had there not been an implementation of a partial curfew on subpopulation 2, our model predicts that 22% of this population would not have been infected (Fig. 4), which is equivalent to averting COVID-19 peak incidence by 62.5% in subpopulation 2 (Fig. 4). In the presence of increasing cross-transmission, the flattening effect of the NPI on P_1_ is compromised, suggesting that strategies curbing the epidemic outbreak in one group may become less effective if cross-transmission is not sufficiently controlled.

## Discussion

We demonstrate how a curfew intervention was associated with a greater negative impact of COVID-19 in non-Kuwaiti populations of lower socio-economic status compared to the Kuwaiti population due to an increased in-house contact rate under curfew. A recent study by Hamadah and colleagues further demonstrated a two-fold increased odds of death and ICU admission in the non-Kuwaiti population when compared to the Kuwaiti population in Kuwait [21]. Socioeconomic and demographic factors such as ethnicity, population density, housing conditions and education level are known to be associated with increased transmission and mortality from viral illness [22–25]. The same trend appears to be manifesting in the COVID-19 pandemic globally, with a disproportionate burden of cases and mortality in lower socioeconomic groups [10–12]. These are the same groups that tend to disproportionately suffer from the unintended consequences of NPIs [26, 27].

Our study is corroborated by the COVID-19 experience in Singapore. A study by Koo and colleagues evaluating a Singaporean population recommended a comprehensive intervention involving quarantine, school closures and workplace distancing to reduce SARS-CoV-2 infection once community transmission was established. They did, however, note that at ${\mathcal{R}}_0>2$, certain dense residential clusters in Singapore showed strong viral persistence despite these measures and importantly, their study did not take into account migrant workers who travel daily from Malaysia to Singapore [28]. Despite very successful efforts at dealing with the initial spread of infection, a second resurgence of infections was challenging for Singapore to cope with as the majority of cases were found amongst migrant workers living in dense dormitories [29].

Another epidemiological study from Wuhan, China demonstrated a reduction in effective reproduction number of SARS-CoV-2 after introduction of quarantine and other measures from above 3·0 at baseline to less than 0·3 after March 1st [30]. Historically, early implementation of multiple interventions are associated with reduced disease transmission and death rates [8]. While there is clear epidemiological rationale supporting quarantine as a measure for limiting viral transmission, the evidence base continues to be weak due to difficulties in carrying out such studies [31]. Some data has suggested that while home quarantine can reduce community spread, it may also lead to an increase in infection of co-habitants [31–33]. In the current epidemic, the attack rate of SARS-CoV-2 in households appears to be in the range of 10-16%; however, little is known about the socioeconomic factors of these households, and we would expect higher attack rates in crowded living conditions [34–36].

In our study, after the institution of a partial lockdown, there was a rise in ${\mathcal{R}}_e$ of non-Kuwaitis as well as a greater variability in the confidence interval. This may be explained by Kuwaitis and non-Kuwaitis of higher socioeconomic status having greater ability to stay at home and institute a measure of physical distancing within their homes. On the other hand, workers who are more likely to live in crowded dormitories were now spending more time in close proximity in indoor environments without wearing masks. Had migrant workers been able to physically distance themselves and effectively practiced universal masking, the incidence of infection could have decreased. However, cohabitants who were shift workers could no longer stagger their time at home as a direct result of the partial lockdown and were forced into closer proximity. This, in addition to threats to food and livelihood security, likely inflamed the situation [37]. Indeed, recent evidence based on spatiotemporal data suggested that the exponential phase of the epidemic started in late March, after the lockdown was in place [38]. The same study highlighted the benefits of introducing spatially heterogeneous interventions whereby additional control measures were localized to the migrant hotspots resulting from the partial lockdown.

Another important observation is the modest decrement in ${\mathcal{R}}_e$ in Kuwaitis after the lockdown. For such an extreme measure with such significant economic connotations, we would have expected a greater effect, such as a reduction of ${\mathcal{R}}_e$ below 1·0, which signals a receding disease transmission among Kuwaitis. This may be explained by the proximity of Kuwaitis and non-Kuwaitis, even throughout the lockdown [24, 25]. Given that many non-Kuwaitis provide essential services to the country, there is a strong possibility that infection was transmitted to Kuwaiti nationals despite lockdown measures in place. It follows from this that disparities within a population lead to worsening of disease transmission for all components of society, a more prolonged pandemic phase and potentially heavier impacts on the economy as a whole. We hypothesize that more equitable societies are likely to fare better (and more predictably) in this pandemic than less equitable ones.

Significant attempts were made to ease the burden of COVID-19 in non-Kuwaiti populations. Healthcare was declared free of charge for non-Kuwaitis with COVID-19 [39], funds were raised to support families and workers affected by the pandemic and field hospitals and quarantine facilities were established in the most densely populated areas. Importantly, the crisis has led to a heightened media focus on human trafficking and labor laws as important culprits in the poor living conditions of workers. This has resulted in a two-pronged government policy: pursue and punish lawbreakers, and provide amnesty and repatriation for workers trapped without valid papers [37, 38, 40]. These attempts came to national attention during the pandemic to help limit disease spread by caring for vulnerable populations and supporting a more general public health framework.

### Limitations

Our modeling results should be interpreted qualitatively rather than taken as projections of the epidemic curve and associated burdens such as mortality. Models are simplifications of a complex reality which, in our case, is concerned with an emergent and complex infectious disease. Tackling such a complexity in a meaningful way requires both a sufficient understanding of the disease dynamics and sustainable access to a range of important datasets. Our analyses, however, were restricted by limitations in data availability which may be related to how data is collected and shared publicly. At the time of writing, concise information on a) daily testing rates, b) random testing, c) contact tracing and isolation, d) location, demographics and clinical manifestation of all cases was not made publicly available. Without such data, our analyses could not dissociate the effect of the infectivity of different modes of the disease from the effectiveness parameter κ. In addition, as the pandemic progressed, information about nationality became more restricted. We were unable to divide non-Kuwaiti cases into those who worked in labor and those who were more skilled with potentially higher socioeconomic status. We believe that our data largely reflects manual workers given their initial clustering in highly dense worker dormitories and areas. Finally, around 750,000 non-Kuwaitis (members of Subpopulation 2) are live-in domestic workers. However, this argues more strongly for our case, as their dilutionary effect likely blunted the calculated rise in ${\mathcal{R}}_e$ amongst P_2_.

Pre-symptomatic and asymptomatic transmissions have been confirmed to play an important role in driving SARS-CoV-2 outbreaks, particularly in geographic areas where case ascertainment and testing rates or scope are low. Physical distancing mandates in such areas are believed to be an important NPI measure to control the progression of the outbreak. However, in our case, the living conditions of the migrant worker subpopulation led to a paradoxical outcome. We have not attempted to explicitly model physical distancing via compartmental subdivision within each subpopulation. Instead we chose parsimony by accounting for such a measure in terms of changes in the contact frequency as captured by the effectiveness parameter *κ*.

While we acknowledge that at the earliest stages of the outbreak, surveillance was mostly symptom-based, we believe that early in the outbreak the contact-tracing capacity in Kuwait may have been sufficiently effective in offsetting this and hence capturing asymptomatic cases. It is also widely acknowledged that a transparent tracking and reporting of data from random testing and contact tracing is vital for quantifying the levels of community transmission, acquired immunity and population interaction. Such unavailable data is key for informing transmission models, particularly when there is uncertainty about the relative importance of the different transmission routes of SARS-CoV-2.

## Conclusions

We demonstrate how lockdown policies can paradoxically facilitate COVID-19 transmission among those who cannot practice physical distancing in non-optimal living conditions. To be effective, interventions intended to promote physical distancing and isolation need to account for existing socioeconomic and health disparities.

## Supplementary Information

**Additional file 1. Supplementary Material.**

## Data Availability

The datasets generated and/or analyzed during the current study are publicly available datasets. Data related to the COVID-19 confirmed cases and deaths in the state of Kuwait are available in an online repository at https://github.com/coronamapskw. Data about population demographics were obtained from the Online Statistics Service System at the Public Authority for Civil Information and are available online in Kuwait only via http://stat.paci.gov.kw/englishbuildreports/. All data and codes that support the findings of this study are fully available from the corresponding author, upon reasonable request.

## Abbreviations

**NPI**: Non-Pharmaceutical Interventions
**SARS-CoV-2**: Severe Acute Respiratory Syndrome – Coronavirus – 2
**COVID-19**: Coronavirus Disease - 2019

## References

[1] World Health Organisation. Coronavirus disease 2019 (COVID-19) Situation Report – 31. Geneva, Switzerland; 2020. https://www.who.int/docs/default-source/coronaviruse/situation-reports/20200220-sitrep-31-covid-19.pdf?sfvrsn=dfd11d24_2. Accessed 24 April 2020.

[2] KUNA. كونا : (الصحة) الكويتية: لم يتم استثناء أحد ممن وصلوا فجر اليوم من (طهران) و (قم) من الحجر الصحي الاجباري - الصحة والبيئة - 24/02/2020. https://www.kuna.net.kw/ArticleDetails.aspx?id=2864468. Accessed 24 Apr 2020.

[3] Tuite AR, Bogoch II, Sherbo R, Watts A, Fisman D, Khan K. Estimation of coronavirus disease 2019 (COVID-19) burden and potential for internationaldissemination of infection from Iran. Ann Intern Med. 2020;172(10):699–701. 10.7326/M20-0696. Epub 2020 Mar 16. 10.7326/M20-0696 PMC7081176 32176272

[4] KUNA : Kuwait gov’t imposes partial curfew, extends holiday for two weeks - MoI - Government - 22/03/2020. https://www.kuna.net.kw/ArticleDetails.aspx?id=2880575&language=en. Accessed 11 May 2020.

[5] KUNA : Kuwait gov’t suspends commercial flights as of midnight March 13 - Government - 11/03/2020. https://www.kuna.net.kw/ArticleDetails.aspx?id=2876451&language=en. Accessed 11 May 2020.

[6] KUNA : Kuwait extends school closure until March 26 - spokesman - Government - 09/03/2020. https://www.kuna.net.kw/ArticleDetails.aspx?id=2874997&language=en. Accessed 11 May 2020.

[7] Hartley DM, Perencevich EN (2020). Public health interventions for COVID-19: emerging evidence and implications for an evolving public health crisis. JAMA..

[8] Hatchett RJ, Mecher CE, Lipsitch M (2007). Public health interventions and epidemic intensity during the 1918 influenza pandemic. Proc Natl Acad Sci U S A.

[9] Legido-Quigley H, Asgari N, Teo YY, Leung GM, Oshitani H, Fukuda K, Cook AR, Hsu LY, Shibuya K, Heymann D (2020). Are high-performing health systems resilient against the COVID-19 epidemic?. Lancet..

[10] White C, Nafilyan V. Coronavirus (COVID-19) related deaths by ethnic group, England and Wales - Office for National Statistics https://www.ons.gov.uk/peoplepopulationandcommunity/birthsdeathsandmarriages/deaths/articles/coronavirusrelateddeathsbyethnicgroupenglandandwales/2march2020to10april2020. Accessed 13 May 2020.

[11] Chung H, Fung K, Ferreira-Legere L, Chen B, Ishiguro L, Kalappa G, et al. COVID-19 Laboratory Testing in Ontario: Patterns of Testing and Characteristics of Individuals Tested, as of April 30, 2020. . COVID-19 Laboratory Testing in Ontario: Patterns of Testing and Characteristics of Individuals Tested, as of April 30, 2020. Toronto, ON: ICES; 2020. https://www.ices.on.ca/Publications/Atlases-and-Reports/2020/COVID-19-Laboratory-Testing-in-Ontario. Accessed 13 May 2020.

[12] Taylor K-Y. The black plague. The New Yorker https://www.newyorker.com/news/our-columnists/the-black-plague. Accessed 13 May 2020.

[13] Twahirwa Rwema JO, Diouf D, Phaswana-Mafuya N, Rusatira JC, Manouan A, Uwizeye E, Drame FM, Tamoufe U, Baral SD (2020). COVID-19 across Africa: epidemiologic heterogeneity and necessity of contextually relevant transmission models and intervention strategies. Ann Intern Med.

[14] Public Authority for Civil Information. Statistics Services System. Stat Designer. 2019. https://www.paci.gov.kw/stat/Default.aspx. Accessed 15 May 2020.

[15] Central Agency for Information Technology. COVID 19 Updates .::. Home. https://corona.e.gov.kw/En. Accessed 23 May 2020.

[16] Al-Shall Report. Kuwaitis, expats wage difference 114.9% in public sector. ARAB TIMES - KUWAIT NEWS. 2019. http://www.arabtimesonline.com/news/kuwaitis-expats-wage-difference-114-9-in-public-sector/. Accessed 11 May 2020.

[17] Ministry of Health, Kuwait. وزارة الصحة - الكويت (@KUWAIT_MOH) / Twitter. Twitter. https://twitter.com/kuwait_moh. Accessed 23 May 2020.

[18] OpenStreetMap. OpenStreetMap. https://www.openstreetmap.org/. Accessed 23 May 2020.

[19] Al-Shammari AAA, Ali H, Al-Ahmad B, Al-Refaei FH, Al-Sabah S, Jamal MH, et al. Real-time tracking and forecasting of the COVID-19 outbreak in Kuwait: a mathematical modeling study. medRxiv. 2020;:2020.05.03.20089771.

[20] Core Team R (2014). R: a language and environment for statistical computing.

[21] Hamadah H, Alahmad B, Behbehani M, Al-Youha S, Almazeedi S, Al-Haddad M (2020). COVID-19 clinical outcomes and nationality: results from a Nationwide registry in Kuwait. BMC Public Health.

[22] Grantz KH, Rane MS, Salje H, Glass GE, Schachterle SE, Cummings DAT (2016). Disparities in influenza mortality and transmission related to sociodemographic factors within Chicago in the pandemic of 1918. Proc Natl Acad Sci U S A.

[23] Quinn SC, Kumar S (2014). Health inequalities and infectious disease epidemics: a challenge for global health security. Biosecur Bioterror.

[24] Chen J, Chu S, Chungbaek Y, Khan M, Kuhlman C, Marathe A, Mortveit H, Vullikanti A, Xie D (2016). Effect of modelling slum populations on influenza spread in Delhi. BMJ Open.

[25] Adiga A, Chu S, Eubank S, Kuhlman CJ, Lewis B, Marathe A, Marathe M, Nordberg EK, Swarup S, Vullikanti A, Wilson ML (2018). Disparities in spread and control of influenza in slums of Delhi: findings from an agent-based modelling study. BMJ Open.

[26] Zargar A. India’s poor hit hardest as coronavirus spreads and lockdown is extended. https://www.cbsnews.com/news/india-coronavirus-covid19-poor-hit-hardest-lockdown-extended-narendra-modi-today-2020-04-14/. Accessed 13 May 2020.

[27] Alahmad B, Kurdi H, Colonna K, Gasana J, Agnew J, Fox MA (2020). COVID-19 stressors on migrant workers in Kuwait: cumulative risk considerations. BMJ Glob Health.

[28] Koo JR, Cook AR, Park M, Sun Y, Sun H, Lim JT, Tam C, Dickens BL (2020). Interventions to mitigate early spread of SARS-CoV-2 in Singapore: a modelling study. Lancet Infect Dis.

[29] Cai W, Lai KKR. Packed With Migrant Workers, Dormitories Fuel Coronavirus in Singapore. The New York Times. 2020. https://www.nytimes.com/interactive/2020/04/28/world/asia/coronavirus-singapore-migrants.html. .

[30] Pan A, Liu L, Wang C, Guo H, Hao X, Wang Q, et al. Association of Public Health Interventions with the epidemiology of the COVID-19 outbreak in Wuhan, China. JAMA. 2020. 10.1001/jama.2020.6130 PMC7149375 32275295

[31] Fong MW, Gao H, Wong JY, Xiao J, Shiu EYC, Ryu S, et al. Nonpharmaceutical measures for pandemic influenza in nonhealthcare settings-social distancing measures. Emerging Infect Dis. 2020;26(5):976–84. 10.3201/eid2605.190995. 10.3201/eid2605.190995 PMC7181908 32027585

[32] Miyaki K, Sakurazawa H, Mikurube H, Nishizaka M, Ando H, Song Y, Shimbo T (2011). An effective quarantine measure reduced the total incidence of influenza a H1N1 in the workplace: another way to control the H1N1 flu pandemic. J Occup Health.

[33] van Gemert C, Hellard M, McBryde ES, Fielding J, Spelman T, Higgins N, Lester R, Vally H, Bergeri I (2011). Intrahousehold transmission of pandemic (H1N1) 2009 virus, Victoria, Australia. Emerg Infect Dis.

[34] Wei WE. Presymptomatic Transmission of SARS-CoV-2 — Singapore, January 23–March 16, 2020. MMWR Morb Mortal Wkly Rep. 2020;69. doi:10.15585/mmwr.mm6914e1. 10.15585/mmwr.mm6914e1 PMC7147908 32271722

[35] Bi Q, Wu Y, Mei S, Ye C, Zou X, Zhang Z, Liu X, Wei L, Truelove SA, Zhang T, Gao W, Cheng C, Tang X, Wu X, Wu Y, Sun B, Huang S, Sun Y, Zhang J, Ma T, Lessler J, Feng T (2020). Epidemiology and transmission of COVID-19 in 391 cases and 1286 of their close contacts in Shenzhen, China: a retrospective cohort study. Lancet Infect Dis.

[36] Li W, Zhang B, Lu J, Liu S, Chang Z, Cao P, et al. The characteristics of household transmission of COVID-19. Clin Infect Dis. 2020. 10.1093/cid/ciaa450 PMC7184465 32301964

[37] 2020. الحطاب خ. 356 ألفاً یقطنون في مساكن مكدسة. جریدة القبس الإلكتروني. https://alqabas.com/article/5764616. Accessed 11 May 2020.

[38] Alkhamis MA, Youha SA, Khajah MM, Haider NB, Alhardan S, Nabeel A (2020). Spatiotemporal dynamics of COVID-19 epidemic in the State of Kuwait. Int J Infect Dis.

[39] KUNA. كونا : سمو أمير البلاد يوجه كلمة إلى إخوانه وأبنائه المواطنين والمقيمين - عام - 22/03/2020. https://www.kuna.net.kw/ArticleDetails.aspx?id=2880748&language=ar#. Accessed 21 May 2020.

[40] Nagraj. Kuwait to allow repatriation flights, offers amnesty for expats without valid residencies. Gulf Business. 2020. https://gulfbusiness.com/kuwait-to-allow-repatriation-flights-offers-amnesty-for-expats-without-valid-residencies/. Accessed 12 May 2020.

