# Supplementary material for: The effects of non-pharmaceutical interventions on SARS-CoV-2 transmission in different socioeconomic populations in Kuwait: a modeling study
Source: BMC Public Health. 2021 May 26;21:990. doi: 10.1186/s12889-021-10984-6 (PMC8152192; doi:10.1186/s12889-021-10984-6)
Supplement: Supplementary file 1 — Additional file 1. Supplementary Material. [file 12889_2021_10984_MOESM1_ESM.docx]

**­APPENDIX**

**Table of contents:**

A) Sociodemographics of non-Kuwaitis residing in Kuwait…………………………….……2
B) Modified metapopulation SEIR model…………………………………………..….……..3
C) Supplementary Figures and Tables………………………………………………....……...4
 1. Table A1 Parameters of the modified metapopulation SEIR model………….…5

2. Figure S1 Modified SEIR model fitted to case data …………………….………6
D) References………………………………………………………………………………….7

## Sociodemographics of non-Kuwaitis residing in Kuwait

Kuwait has a population of approximately 4.8 millions of which 70% are non-Kuwaiti [1]. Kuwait’s visa regulations are based on a labor mobility system where visas are offered to Temporary Contractual Workers (TCWs) [2]. TCWs include skilled workers, unskilled workers and domestic help. The majority of non-Kuwaitis are from Asia, with most coming from India. The next largest group are Arabs, of which most are from Egypt. Put together, these two nationalities make up more than 50% of TCWs [3]. Additionally, there are at least 100,000 stateless individuals residing in Kuwait who classified as non-Kuwaitis and who have limited health and employment opportunities [4]. The profile of the Non-Kuwaiti population is predominantly male (69%), poorly educated (68% below secondary level education) and relatively young (median age group 30-34) (See Table 1 in main manuscript and Fig. A1 below). The income level of a male worker determines their ability to sponsor family members for residency in Kuwait while female TCWs are largely not able to, regardless of income. Most foreign laborers being in the service industry are unlikely to meet these conditions and thus live the life of a bachelor [3].

Residential areas where non-Kuwaitis live are more likely to consist of apartment units rather than houses (Fig. 2 in main manuscript). It is estimated that there are 1,237,677 males living in around 184,486 units around the country. Almost a million are spread out across eight densely populated areas [5]. Two of the most densely populated areas are home to 356,157 men, where the vast majority live in shared accommodation. One small two-bedroom apartment can typically house 8-10 individuals and sometimes more [6, 7].

All TCWs in Kuwait are tied to an employer that sponsors them through the ‘Kafala’ system. This system prevents them from changing jobs or leaving the country without their employer’s permission. Without the ability to terminate the employment contracts at will, this system leaves significant gaps for exploitation [2]. Indeed, over decades, the country has accumulated an underground network of front companies and powerful individuals who have exploited the Kafala system to traffic workers. There are no accurate numbers on the number of workers exploited in this way but estimates put them to be over 400,000[8]. Health access is also more limited for non-Kuwaitis, who are largely expected to pay out-of-pocket for healthcare [2, 9]. Finally, TCWs are also not covered by national social security [2, 10].

## Modified Metapopulation SEIR model

Our modified metapopulation SEIR model divides the population into two distinct socioeconomic groups: Subpopulation 1 (P_1_) of higher socioeconomic status and Subpopulation 2 (P_2_) of lower socioeconomic status. We analyze the disease transmission by using a modified SEIR model that describes the epidemiological characteristics of COVID-19. The model divides individuals within each subpopulation into the following infection classes or compartments: susceptible (S), exposed but not infectious (E), asymptomatic infectious (I_A_), pre-symptomatic infectious (I_P_), symptomatic infectious (I_S_), and removed (R). The progression through the different compartments is described by key durations that are known to characterize the infection transmission dynamics of COVID-19 (see model parameters in Table A1 below). Transmission is possible from symptomatic cases (clinical), pre-symptomatic cases (pre-clinical) and asymptomatic cases (subclinical). We also describe the interaction between the two subpopulations using two adjustable parameters ($\beta_{12}$ and $\beta_{21}$) to model the successful transmission occurring upon cross-contact between individuals of P_1_ and P_2_. These cross-transmission parameters are typically used to model the probabilities of successful contacts between individuals across a network of metapopulations.

For a given subpopulation *i*, we describe the epidemic transmission by the following system of ordinary differential equations

$$\frac{dS_{i}}{dt}= -S_{i}\sum_{j} \beta_{ij}\left( \frac{I_{Pj}+I_{Sj}+\delta I_{Aj}}{N_{j}} \right)$$

$$\frac{dE_{i}}{dt}= S_{i}\sum_{j} \beta_{ij}\left( \frac{I_{Pj}+I_{Sj}+\delta I_{Aj}}{N_{j}} \right)-\sigma E_{i}$$

$$\frac{dI_{Pi}}{dt}= \left( 1-f \right)\sigma_{i}E_{i}-\gamma_{p}I_{Pi}$$

$$\frac{dI_{Ai}}{dt}= f\sigma_{i}E_{i}-\gamma_{a}I_{Ai}$$

$$\frac{dI_{Si}}{dt}= \gamma_{p}I_{Pi}-\gamma_{s}I_{Si}$$

$$\frac{dR_{i}}{dt}=\gamma_{s}I_{Si}+\gamma_{a}I_{Ai}$$

where $i, j=1,2$, $\beta_{ij}$ describes the transmission rate from subpopulation $j$ to $i$, $1/\sigma$ is average latent duration, and $1/\gamma_{p}$ is the average pre-symptomatic infectiousness period, $1/\gamma_{s}$ is the average symptomatic infectiousness period, $1/\gamma_{a}$ is the average asymptomatic infectiousness period, $\delta=0.5$ is the relative infectiousness of asymptomatic cases compared to symptomatic ones, $f=0.2$ is the proportion of exposed individuals developing an asymptomatic form of the infection, and $N_{j}$ is the size of subpopulation *j*. The outbreak is simulated by using the parameters presented in Table A1.

Based on the assumption of an early decoupled transmission dynamics between the two subpopulations, we compute the basic reproduction number for each subpopulation by reducing the metapopulation model to a single-population modified SEIR model. Using a next generation matrix approach, we derive the following formula for the basic reproduction number of this reduced model

$$\mathcal{R}_{0}=\frac{\beta}{\gamma_{a}}\left( \delta f \right)+\frac{\beta}{\gamma_{p}}\left( 1-f \right)+\frac{\beta}{\gamma_{s}}\left( 1-f \right),$$

where $\beta=\beta_{ii}$ is the within subpopulation transmission rate. We assume that $\beta=\kappa\beta_{0}$, where $\beta_{0}$ is a baseline transmission rate characterizing $\mathcal{R}_{0}$ and $\kappa$ is a scaling factor characterizing the effectiveness of the implementation of a nationwide partial lockdown in Kuwait on 22 March 2020. As described in the main manuscript, we estimate this effectiveness parameter before and after the implementation of the partial lockdown by fitting the model to daily reported case numbers.

1. **Supplementary Figures and Tables**

**Table A1. Parameters of the modified metapopulation SEIR model**

| **Parameters** | **Symbol** | **Values** | **Justification** |
| --- | --- | --- | --- |
| Average incubation period |  | 3.5 days | [11–13] |
| Average latent period | $1/\sigma$ | 2.5 days | [11–13] |
| Average pre-symptomatic infectiousness period | $1/{\gamma_{p}}$ | 1 day | [11–13] |
| Average symptomatic infectiousness period | $1/{\gamma_{s}}$ | 6 days | [11–13] |
| Average asymptomatic infectiousness period | $1/{\gamma_{a}}$ | 7 days | [11–13] |
| Transmission rates | $\beta_{12}$ | 0 – 0·02* | Assumed |
|  | $\beta_{21}$ | 0 – 0·02* | Assumed |
|  | $\beta_{11}$ | 0.171** | Estimated |
|  | $\beta_{22}$ | 0.374** | Estimated |
| Proportion of asymptomatic individuals | $f$ | 20% | [14] |
| Relative infectiousness of asymptomatic cases | $\delta$ | 50% | [11–13] |

* Adjustable parameters for model scenarios.
** Effective transmission rate over the entire period.

**B**

**A**

**Figure S1. Modified SEIR model fitted to case data.** (A) Fitted model for case data of Subpopulation 1. (B) Fitted model for case data of Subpopulation 2. Note how the outbreak in subpopulation 1 remained under control, while in subpopulation 2 the numbers increased post-lockdown. Grey discs represent all daily reported case data (symptomatic and asymptomatic). Black dashed curve represents the modified SEIR model projection. The 95% CI of this projection is shaded in the red color. The fitting was carried out for the period from 25/2/20 to 19/4/2020 as detailed in the main manuscript
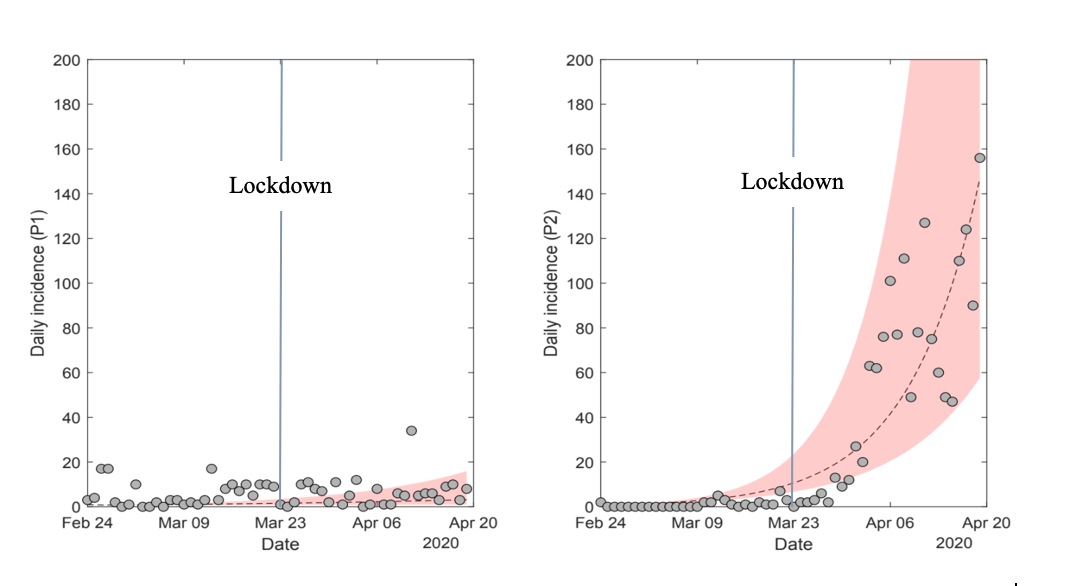
.

**References**

1. Public Authority for Civil Information. Statistics Services System. Stat Designer. 2019. https://www.paci.gov.kw/stat/Default.aspx. Accessed 15 May 2020.

2. International Organization for Migration. Migration Governance Overview: The State of Kuwait. 2018. https://migrationdataportal.org/sites/default/files/2019-09/MGI%20Kuwait%20Report%20FINAL.pdf. Accessed 24 Apr 2020.

3. De Bel-Air F. The Demographic and Economic Framework of Migration in Kuwait. https://cadmus.eui.eu/bitstream/handle/1814/32155/GLMM%20ExpNote_01-2013.pdf. Accessed 24 Apr 2020.

4. Albloshi HH. Stateless in Kuwait. Arab Gulf States Institute in Washington. 2019. https://agsiw.org/stateless-in-kuwait/. Accessed 24 Apr 2020.

5. Al-Saqabi A. «كوتا» للجنسيات.. محاربة تجار الإقامات .. دعم العمالة الوطنية. جريدة القبس الإلكتروني. 2020. https://alqabas.com/article/5767918. Accessed 24 Apr 2020.

6. الحطاب خ. 356 ألفاً يقطنون في مساكن مكدسة. جريدة القبس الإلكتروني. 2020. https://alqabas.com/article/5764616. Accessed 11 May 2020.

7. الزيد ج, سالم ع. مأساة 14 مقيماً ضحية «تجار الإقامات».. يسكنون في شقة صغيرة. لقبس الإلكتروني. https://alqabas.com/watch/5767848. Accessed 11 May 2020.

8. Alqabas. محمد الصقر يعلّق على قضية تجارة الإقامات: العمالة الهامشية.. 400 ألفٍ. جريدة القبس الإلكتروني. 2020. https://alqabas.com/article/5769490-. Accessed 11 May 2020.

9. Health fee increase for expats in Kuwait. ARAB TIMES - KUWAIT NEWS. 2019. http://www.arabtimesonline.com/news/health-fee-increase-for-expats-in-kuwait/. Accessed 11 May 2020.

10. The Public Institution for Social Security. Social Security Guide in Kuwait. 2009. https://www.pifss.gov.kw/upload/pifss_e_guide_eng_212.pdf. Accessed 25 Apr 2020.

11. Bi Q, Wu Y, Mei S, Ye C, Zou X, Zhang Z, et al. Epidemiology and transmission of COVID-19 in 391 cases and 1286 of their close contacts in Shenzhen, China: a retrospective cohort study. Lancet Infect Dis. 2020;20:911–9.

12. Tindale LC, Stockdale JE, Coombe M, Garlock ES, Lau WYV, Saraswat M, et al. Evidence for transmission of COVID-19 prior to symptom onset. eLife. 9. doi:10.7554/eLife.57149.

13. Wölfel R, Corman VM, Guggemos W, Seilmaier M, Zange S, Müller MA, et al. Virological assessment of hospitalized patients with COVID-2019. Nature. 2020;581:465–9.

14. Buitrago-Garcia D, Egli-Gany D, Counotte MJ, Hossmann S, Imeri H, Ipekci AM, et al. Occurrence and transmission potential of asymptomatic and presymptomatic SARS-CoV-2 infections: A living systematic review and meta-analysis. PLOS Med. 2020;17:e1003346.

**End of Appendix**
